# [Retracted] Expression of α-fetoprotein in gastric cancer AGS cells contributes to invasion and metastasis by influencing anoikis sensitivity

**DOI:** 10.3892/or.2025.8945

**Published:** 2025-07-14

**Authors:** Sumei Lu, Yongmei Ma, Tao Sun, Rui Ren, Xiaoning Zhang, Wanshan Ma

Oncol Rep 35: 2984–2990, 2016; DOI: 10.3892/or.2016.4678

Following the publication of this paper, it was drawn to the Editor's attention by a concerned reader that several instances of duplicated data existed within several of the figures in this article; specifically:

i) two pairs of data panels in the four-panel Fig. 2B (so affecting all the panels in this figure part, which showed the results from migration and invasion assay experiments) were overlapping;

ii) a pair of data panels for the fluorescence experiments shown in Fig. 4 were also found to be overlapping;

and iii) one set of protein bands in the western blots featured in Fig. 5 (for the caspase-3 data) had also been included in a paper submitted to and published in *Oncology Reports* a few months earlier, with the author Tao Sun held in common between these papers.

Owing to the number of cases of data duplication that were identified in this paper, the Editor of *Oncology Reports* has decided that it should be retracted from the Journal on account of a lack of confidence in the presented data. After contacting the authors, they accepted the decision to retract the paper. The Editor apologizes to the readership for any inconvenience caused.

